# Integrative transcriptomics reveals genotypic impact on sugar beet storability

**DOI:** 10.1007/s11103-020-01041-8

**Published:** 2020-08-04

**Authors:** Silvia Madritsch, Svenja Bomers, Alexandra Posekany, Agnes Burg, Rebekka Birke, Florian Emerstorfer, Reinhard Turetschek, Sandra Otte, Herbert Eigner, Eva M. Sehr

**Affiliations:** 1 AIT Austrian Institute of Technology, Center for Health & Bioresources, Tulln, Austria; 2 Center for Integrative Bioinformatics Vienna, Max Perutz Labs, University of Vienna, Medical University of Vienna, Vienna, Austria; 3 University of Technology Vienna, Research Unit of Computational Statistics, Vienna, Austria; 4 AGRANA Research & Innovation Center GmbH, Tulln, Austria; 5 Strube Research GmbH & Co. KG, Söllingen, Germany

**Keywords:** Storability, Sugar beet, Transcriptomics, Anatomy, Sugar analytics, Post-harvest storage

## Abstract

**Key message:**

An integrative comparative transcriptomic approach on six sugar beet varieties showing different amount of sucrose loss during storage revealed genotype-specific main driver genes and pathways characterizing storability.

**Abstract:**

Sugar beet is next to sugar cane one of the most important sugar crops accounting for about 15% of the sucrose produced worldwide. Since its processing is increasingly centralized, storage of beet roots over an extended time has become necessary. Sucrose loss during storage is a major concern for the sugar industry because the accumulation of invert sugar and byproducts severely affect sucrose manufacturing. This loss is mainly due to ongoing respiration, but changes in cell wall composition and pathogen infestation also contribute. While some varieties can cope better during storage, the underlying molecular mechanisms are currently undiscovered. We applied integrative transcriptomics on six varieties exhibiting different levels of sucrose loss during storage. Already prior to storage, well storable varieties were characterized by a higher number of parenchyma cells, a smaller cell area, and a thinner periderm. Supporting these findings, transcriptomics identified changes in genes involved in cell wall modifications. After 13 weeks of storage, over 900 differentially expressed genes were detected between well and badly storable varieties, mainly in the category of defense response but also in carbohydrate metabolism and the phenylpropanoid pathway. These findings were confirmed by gene co-expression network analysis where hub genes were identified as main drivers of invert sugar accumulation and sucrose loss. Our data provide insight into transcriptional changes in sugar beet roots during storage resulting in the characterization of key pathways and hub genes that might be further used as markers to improve pathogen resistance and storage properties.

**Electronic supplementary material:**

The online version of this article (10.1007/s11103-020-01041-8) contains supplementary material, which is available to authorized users.

## Introduction

The centralization of sugar factories in Europe is causing an extended processing campaign that makes storage of sugar beet up to 90 days inevitable (Huijbregts et al. 2013). Roots in general show very low storage ability (storability) because of their quite active metabolism that uses sucrose as its energy source (Afek and Kays 2010). The resulting sucrose loss is especially disadvantageous for the sugar industry. Furthermore, the hydrolyzation of sucrose results in the accumulation of invert sugar (glucose and fructose), another negative effect for the sugar industry reducing the efficiency of the sugar manufacturing process (Draycott 2006; Klotz and Finger 2004).

Many factors have already been described to affect the storability of sugar beet. One of the most influential factors are the storage conditions themselves (e.g. temperature, relative humidity) (Kenter and Hoffmann 2009; Klotz and Finger 2004), but severe injuries caused by mechanical harvesting and topping also play a major role in this context (Wiltshire and Cobb 2000). Wounds serve as entry points for microbes into the beet root. Their colonization and activity leads to storage rot and mold, and a temperature-dependent prevalence for the three main associated pathogens, *Botrytis*, *Fusarium* and *Penicillium*, was described (Liebe et al. 2016). To combat the infestation, even more metabolic activity by the beet roots starts a vicious cycle, resulting in higher sucrose metabolization by both plant and microbes, generation of heat, and further spread of pathogens, followed by substantial sucrose yield losses (Campbell and Klotz 2007b; Strausbaugh 2018; Kusstatscher et al. 2019). Intriguingly, some studies found a genetic contribution to storage, with van Swaaij and Huijbregts (2010) describing significant differences in sucrose loss between 12 genotypes and a correlation to initial sucrose content. Similar results by Schnepel and Hoffmann (2016) found genotypic differences in storage loss that seemed to occur mainly based on a different (genotype-specific) microbial composition. It is also known that pathogen resistant genotypes are equipped with better storability (Strausbaugh et al. 2009) and that there is a genotype-specific pathogen profile during storage (Liebe and Varrelmann 2016).

In this context, increased resistance to pathogens was shown to be highly and positively correlated with morphological and anatomical differences like an overall root stability, a specific cell wall composition, and a higher marc content (representing insoluble cell wall components) (Hoffmann et al. 2018; Schnepel and Hoffmann 2016). The amount and composition of cell wall material defines its strength and stability to serve as nonspecific resistance to pathogens (Smirnova and Kochetov 2016; Hoffmann et al. 2018). This is supported by the differential expression of genes after a pathogen attack that are related to cell wall biogenesis, defense, stress, and degradation (Bellin et al. 2007). Also here, a genotypic effect was described insofar as genotypes with high marc concentrations (insoluble cell wall material) before storage showed lower invert sugar accumulation and less infestations with pathogens during storage, yielding better storability (Schnepel and Hoffmann 2016).

It is clear that storage losses cannot be completely prevented but only reduced. However, most of the contributing factors can only be influenced -if at all- to a limited extent. Although the genotypic effect on sucrose loss and accumulation of invert sugar is reportedly low, with 11 and 12%, respectively (Schnepel and Hoffmann 2014), this effect is hypothesized to increase with increasing storage time (Kenter and Hoffmann 2009). Genotype-specific molecular mechanisms associated with extended storage time have not been described so far. Thus, our study focused on the comparative transcriptomics of different sugar beet varieties to find mechanisms that can explain the differing storability potential. We further characterized beet root anatomy, sugar and standard analyte concentrations to identify additional influencing factors. With our results we aimed at providing a knowledge base that targets breeding programs for improved pathogen resistance and storage properties. Both are complex traits that are thought to be more important for a successful sugar beet production than further increasing the yield potential (Hoffmann and Kenter 2018).

## Methods

### Study design

**Fig. 1:**
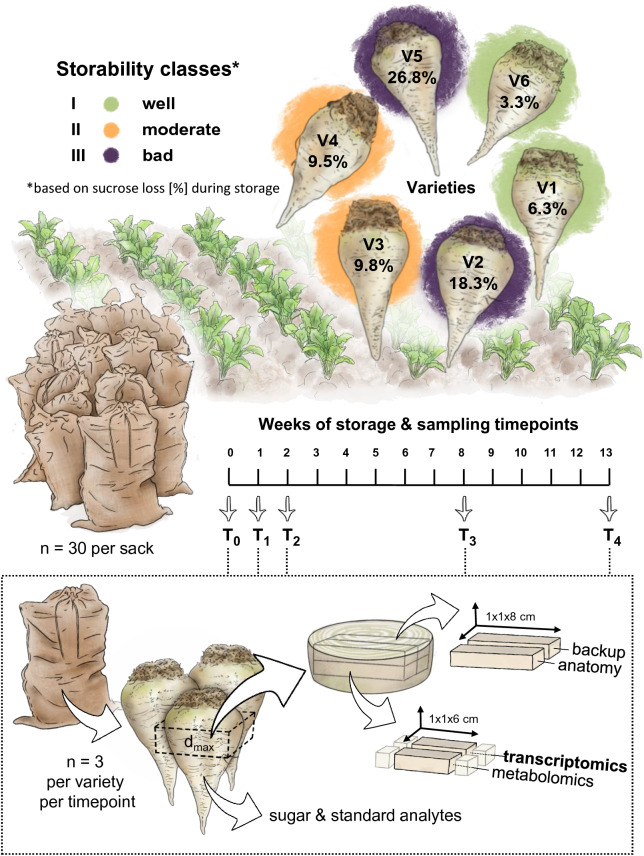
Study design. Six sugar beet varieties with different storability potential based on their relative sucrose loss normalized to the average weight loss (in percent) were grown in a randomized block design, mechanically harvested and stored in sacks (30 beets per sacks) under controlled conditions for 13 weeks. Sampling was done at five timepoints (T0–T4) whereby three individuals per variety were processed. A cross section was cut out at the thickest part of the root, surface sterilized, and four blocks were extracted: for transcriptomics, metabolomics, anatomical analysis, and one as backup. The remaining parts of the root were used for the measurement of sugars and standard analytes

The thereafter described study design is also depicted in Fig. 1: Six different varieties (V1–V6) of sugar beet (*Beta vulgaris* L.) known to have different storability characteristics were included in this study. Varieties V1 to V5 were bred and kindly provided by Strube Research GmbH & Co. KG., Germany, and V6 was kindly provided by AGRANA Research and Innovation Center (ARIC), Austria. All six varieties are Rizomania tolerant, whereby V1 and V6 have an additional Cercospora tolerance, and V1 and V4 are nematode tolerant. All varieties were grown in a randomized plot design (4 reps) in Frauenkirchen, an irrigated site in Austria, in spring 2017 and in 2018 as a backup, managed by ARIC. On 16th October 2017, beets were harvested mechanically with a self-propelled single-row beet harvester. Homogenous samples (with 30 beets per sack) were formed for immediate analysis of sugar and standard analytes (4 reps) and stored (9 reps) under constant conditions (temperature between 13 and 5 °C, whereby the outside temperature was followed to mimic a more realistic storage condition; air humidity between 62 and 76%) in a cooling chamber at ARIC.

The varieties were grouped in three storability classes based on their relative sucrose loss after 13 weeks of storage, measured during the storage trial in 2017 (see Table S1), namely into well (V1 & V6), moderately (V3 & V4), and badly storable (V2 & V5) varieties. A classification, that is supported by data from past storage trials at ARIC (data not shown). In detail, storability was determined by relative sucrose loss (normalized to the average weight loss) over storage time. For this, each sack containing 30 beets was weighed at T0 and at the corresponding sampling time to calculate weight loss for each variety. The average sucrose content (SC) was measured based on the following available replicates (sacks): four sacks at harvest (T0, n = 120), one sack each after one week (T1, n = 30), two weeks (T2, n = 30), six sacks (except V6, five sacks) after eight weeks (T3, n = 180) and one sack after 13 weeks of storage (T4, n = 30). According to the International Commission for Uniform Methods of Sugar Analysis (ICUMSA), beets of each sack were homogenized to beet brei and SC was determined by measuring polarization as °Z (ICUMSA Method GS 6–3, 1994). Average sucrose loss per variety and sampling time was then calculated and normalized to the overall average weight loss (3.26%) at the corresponding sampling time (Table S1).

Sampling for subsequent transcriptomic, metabolomic and anatomical analyses was done at five timepoints: at harvest (16th Oct., T0), 1 week (24th Oct., T1, 91 degree days), 2 weeks (31st Oct., T2, 168 degree days), 8 weeks (12th Dec., T3, 525 degree days), and 13 weeks (16th Jan. 2018, T4, with 704 degree days measured on 12th Jan.) after the harvest. At each timepoint, three individual beets per variety were taken out of one of the sacks as biological replicates, except at T0 (prior to storage), where additionally 10 individuals were taken. After weighing and photographing each individual beet root, a disk was cut out from the middle of the beet root where the root was thickest, and the surface was sterilized with 70% ethanol. Using a French fry cutter, four small rectangular blocks (1 × 1 × 8 cm) were cut out from the slice covering the entire cross-section. Three of the blocks were immediately frozen in liquid nitrogen for downstream transcriptomics and metabolomics, respectively, as well as for having a backup block. Finally, one block was put into the fixative FAA (10 ml 37% formaldehyde, 50 ml 96% ethanol, 5 ml acetic acid, 35 ml water) for one day including a deaeration of 10 min, followed by an ethanol washing series down to 70%, in which the block was kept until further processing for anatomical analysis. The remaining parts of the beet were used for the measurement of sugar and standard analytes at ARIC.

### Beet root anatomy

Per individual, the following parameters were measured: number of cambial rings, periderm thickness, parenchyma cell number and cell area in the six different varieties (with three replicates each) at T0 and T4. Cambial rings were counted simply using the non-processed blocks, that were stored in 70% ethanol. To analyze the periderm and parenchyma tissue, polyethylenglycol (PEG) embedding and micro-cutting of the block samples were done according to the protocol of Gierlinger et al. (2012). Cross Sections (5 µm) were obtained using a Leica rotary microtome, transferred by a scotch tape (tesapack**®** kristall-klar), stained with astra blue and safranin (1:1) and mounted on glass slides. Analysis was done using a Leica light microscope CTR 6000 equipped with a Leica DMC2900 camera and Leica Application Suite X (LAS X) software platform. For estimating the periderm parameters, cell number and cell area analysis of parenchyma tissue, the sections were observed with a × 10 and × 5 magnification lens, respectively. Adobe photoshop version CS2 was used for the selection of the periderm and a section with a defined size of the parenchyma tissue between the last and second last cambial ring. The conversion from pixel into micrometer was calculated using the latest version of ImageJ (Schneider et al. 2012), whereby a conversion factor of 1.16 for a × 5 magnification lens and 0.58 for a × 10 magnification lens was applied.

### Lignin content

The cell wall preparation and lignin quantitation were done according to a minor modified version of the acetyl bromide method described by Moreira-Vilar et al. (2014). The material was prepared as follows: From each side of each individual block the outer layer (representing the epidermis) was cut off (approx. 1.5 cm) and ground. The amount of protein-free cell wall material obtained from around 600 mg of frozen (− 80 °C) ground material was noted. For solubilization of the lignin extract, 4 ml of a solution containing 1.11 ml of 2 M NaOH, 0.12 ml of 5 M hydroxylamine-HCl and 2.77 ml of glacial acetic acid was mixed with the samples. For spectrophotometric measurements, the samples and standards were diluted 20 times inside the cuvettes with the same solution as described above. A standard curve was generated with alkali lignin and the obtained absorptivity value of 21.67 mg^−1^ ml cm^−1^ (also known as M^−1^ cm^−1^, the molar absorption coefficient) was taken to calculate the acetyl bromide soluble lignin (ABSL) concentration using Beer-Lambert law (Pace et al. 1995). The results were expressed as percentage of dried cell wall material. Measurements were done with up to three technical replicates per sample and the average lignin content for the individual beet was calculated. Values outside of the interquartile range (IQR) for each variety and each sampling time were excluded.

### Sugar and standard analytes

On a total of 90 individual beet roots the measurement of sugar and standard analytes was done according to ICUMSA. The remaining parts from the sampling (see Study design) of each individual were homogenized to a beet brei, which was further processed as follows: Sucrose content (SC) was determined by measuring polarization as °Z (ICUMSA Method GS 6-3, 1994) and is hereafter depicted as percentage. Glucose and fructose content [mg/kg] was analyzed enzymatically (ICUMSA Method GS 8/4/6-4, 2007), and summarized as the variable invert sugar. Potassium and sodium [mmol/kg] was analyzed by flame photometry (ICUMSA Method GS 6-7, 2007). Alpha-amino nitrogen [mmol/kg] was analyzed by the copper method (ICUMSA Method GS 6-5, 2007). Marc content was determined by extracting beet pulp with 500 ml of 70 °C hot deionized water in a frit. Insoluble remains (marc content) were dried in an oven (105 °C) and thereafter weighed gravimetrically.

### Descriptive statistics and regression modelling

Descriptive statistical analysis was done using R version 3.6.2 (R Core Team 2018). Plots were generated using the R package ggplot2 (Wickham 2009). A correlogram with Pearson’s rank correlation coefficients of the sugar and standard analytes concentrations of all 90 individual samples and of the anatomy parameters of the 36 individuals was performed with the R package ggcorrplot (Kassambara 2019).

A regression tree analysis was performed to find the most important explanatory factors among the measured sugar parameters, standard analytes, and anatomy-related parameters that correlate with storability. After the exclusion of highly correlated variables from the model, explaining the storability classified in three groups with the independent variables invert sugar, Pol, K + Na, alpha-amino N, marc content, lignin content, periderm thickness, cambial rings, parenchyma cell area, and root weight. Calculation was performed with R package rpart (Therneau and Atkinson 2019; Breiman et al. 1984) using default parameter and visualization was done with package partykit (Hothorn and Zeileis 2015).

### Transcriptomics

Before RNA extraction, the frozen block (see Fig. 1) was prepared as follows: from each side of each individual block the outer layer (representing the epidermis) was cut away (ca. 1.5 cm) and the remaining inner part was further ground using liquid nitrogen with mixing the powder thoroughly.

### RNA extraction, library preparation and sequencing

Per individual sample, around 150 mg of the powder was used for RNA extraction. In case of the additionally sampled 10 individuals per variety at timepoint 0, an equal amount of each individual powder was mixed together to form a variety-specific pool sample. RNA extraction was done on 96 samples in total using TRIzol Reagent following the manufacturer's protocol (Thermo Fisher Scientific Inc.). Finally, the RNA was dissolved in 0.1% DEPC water. Total RNA on dry ice was sent to VBCF NGS Unit. There, the RNA was quality and quantity checked using Agilent’s Bioanalyzer. The library preparation was done with the SENSE mRNA-Seq Library Prep Kit (Lexogen GmbH) and sequencing was performed with Illumina HiSeqV4 with eight samples per lane and as 125 bp paired-end reads.

### Quality control and pre-processing

Raw sequenced reads of all 96 samples were pre-processed with BBDuk (BBMap package, Bushnell 2019a, version 37.90) to guarantee high quality (HQ) reads for further processing. Low quality reads and known Illumina adapters were trimmed. First nine bases were clipped off due to recommendations described in the mRNA-Seq Library Prep Kit V2 User Guide (Lexogen GmbH) and short reads (< 50 bp) were removed. Filtering parameters were set in consideration of the BBDuk guideline (Bushnell 2019b). Quality of raw and preprocessed reads was analyzed with FASTQC version 0.11.5 (Andrews 2010) and MULTIQC version 1.7 (Ewels et al. 2016).

### Mapping and abundance estimation

Mapping of the HQ paired-end reads to the reference genome of *Beta vulgaris* L. (RefBeet-1.2.2), downloaded from Ensembl Plants release 40 (Zerbino et al. 2018), was performed using the splice-aware aligner HISAT2 version 2.1.0 (Kim et al. 2015). HISAT2 was executed with additional parameters indicating strand specificity and known exons and splice sites extracted from the reference annotation file. Quality of the mapping results was investigated with Qualimap version 2.2.1 (Okonechnikov et al. 2016). Abundance estimation of annotated genes was performed with featureCounts (Liao et al. 2014) using paired-end mode and strand-specification.

### Analysis of unmapped reads

In order to identify possible viral and fungal infestation, unmapped reads were rRNA filtered with sortmerna (Kopylova et al. 2012) and de novo assembled for each individuum using Trinity with default values (Grabherr et al. 2011). Pooled samples of T0 were not included in this analysis. Annotation of de novo assembled transcripts were done using BLASTN version 2.9.0 (Camacho et al. 2009) against NCBI nt database (downloaded from ftp://ftp.ncbi.nlm.nih.gov/blast/db/, 2019-08-05) with an e-value threshold of 10^5^, max_target_seqs = 1 and max_hsps = 1. Hits less than 50 nucleotides were removed. Annotations were counted per gene rather than transcripts. Further, results were filtered based on taxonomy, NCBI:txid10239 for viruses and NCBI:txid4751 for fungi.

### PCA

PCA was performed using function plotPCA of R package DESeq2 (Love et al. 2014) with the variance stabilizing transformed (VST) count data. The top 500 genes (highest row variance) were used for this analysis. Examining the results showed four outlier samples (see Fig. S3b) that were excluded from further differentially expressed genes (DEG) and downstream analysis.

### Differential gene expression analysis

Differential gene expression analysis was performed using DESeq2′s Wald test followed by log fold change (logFC) shrinkage (Love et al. 2014). Significantly differentially expressed genes (DEG) were defined if |logFC > 1| and adjusted p-value (padj) < 0.01. Annotation information, including GO terms and *Arabidopsis thaliana* homologs were retrieved from Ensemble database and gene description additionally from NCBI using R packages BiomaRt (Durinck et al. 2005) and rentrez (Winter 2017). KEGG pathways (Kanehisa et al. 2017) were linked with function getGeneKEGGLinks from R package limma (Ritchie et al. 2015).

To find DEGs that are differentially expressed after 13 weeks of storage (T4) in comparison to the time of harvest (T0) commonly in all varieties, DEGs were first computed separately for each variety and only common DEGs (intersection of DEGs for all varieties) were defined as significantly differentially expressed between T4 and T0. To define a single padj-value and logFC value for each gene, the median of all six varieties was computed. To find DEGs between well and badly storable varieties at a certain point in time, a pairwise comparison between each good and each bad variety (V1 vs V2, V1 vs V5, V6 vs V2, and V6 vs V5) was performed. Only the intersection of these found DEGs were defined as significantly differentially expressed between well and badly storable varieties. To define a single padj-value and logFC value the median logFC of all four combinations (V1 vs V2, V1 vs V5, V6 vs V2, and V6 vs V5) was computed. The analysis was not performed directly comparing all samples belonging to well compared to badly storable samples, to increase accuracy in finding DEGs truly associated with storability rather than DEGs associated with only one specific variety.

### GO enrichment

Functional overrepresentation of a set of genes was computed with the Fisher’s exact test using R package topGO (Alexa and Rahnenfuhrer 2019) and further visual representation was done with REVIGO (Supek et al. 2011). GO terms were considered as significantly enriched if p-value < 0.01.

### KEGG pathway analysis

For KEGG pathway analysis (Kanehisa et al. 2017) only DEGs were used. Computation was done using R package KEGGprofile (Zhao et al. 2019) and the function find_enriched_pathway was used to find significantly enriched pathways based on hypergeometric tests. KEGG pathways were considered as significantly enriched if padj < 0.01. For visualization of differentially expressed genes in the pathways, the sum of logFC values was visualized in the pathway plots using package pathview (Luo and Brouwer 2013).

### Weighted gene co-expression network analysis (WGCNA)

WGCNA was performed using DESeq2-computed variance stabilizing transformation (VST) count data with R package WGCNA version 1.68 (Langfelder and Horvath 2008) following the tutorial (Langfelder and Horvath 2016). Initially, genes were filtered for low expressed values if the normalized counts were not higher than five in at least three samples. For the soft-threshold power the value 13 was chosen because it was the lowest number where the scale free topology fit index reached 0.8. Modules were merged with a cutoff of 0.2. We determined for each gene in the most significant module to the specific trait (invert sugar and sucrose loss, respectively), the correlation to the corresponding module eigengene (= module membership, kME) and the gene significance for the specific trait (Pearson’s correlation, GS1). The connectivity degrees of known protein–protein interactions (PPI) were computed using known STRING interactions of *Beta vulgaris* (NCBI:txid161934) with a confidence value higher than 0.5 (Szklarczyk et al. 2019). For the computation of the PPI connectivity degree highly associated to invert sugar, all genes/proteins of modules pink and midnightblue were included; for the PPI connectivity degree highly associated to sucrose loss, all genes/proteins in the modules green and lightpink4. To define hub genes, following thresholds were set: GS1 > 0.6, kME > 0.8, intramodular connectivity of WGCNA (kWithin) scaled by maximum value in associated module > 0.7, PPI connectivity degree for invert sugar ≥ 10, PPI connectivity degree for sucrose loss ≥ 40.

## Results

### Sucrose content and losses

Considering the sack data (Table S1), before storage at T0, V6 showed the highest sucrose content (SC) value (16.84%), followed by V3 (16.48%). The remaining varieties had a similar SC at T0 varying between 14.14 and 14.71%. After 13 weeks of storage (T4), V6 and V3 had the highest SC, with 16.84 and 15.36% respectively, and V2 and V5 were characterized with the least SC, 12.41 and 10.70%, respectively.

In our study, the definition of storability is based on the variety-specific sucrose loss: V2 and V5 showed the highest loss rate with 18.33 and 26.81%, respectively, and were considered as bad storable. V3 and V4 were considered as moderate (intermediate storability), with a loss rate of 9.81 and 9.51%, respectively, in comparison to V1 and V6, the two varieties that kept the SC relatively constant with a loss rate of 6.33 and 3.28%, respectively, which were thus defined as the two well storable varieties (Fig. 1, Table S1). Since in correlation, sucrose loss was accompanied by an increase of invert sugar during storage for all varieties, from an average of 1357 mg/kg at T0 to an average of 3935 mg/kg at T4 (Table S2). Interestingly, already before storage, the badly storable varieties had a higher content of invert sugar (a median of 1292 mg/kg compared to 734 mg/kg in well storable varieties), the difference was even more distinct at T4 (with a median of 5804 mg/kg compared to 2263 mg/kg). With regard to the marc content, good storable varieties had a higher marc content than badly storable varieties prior to storage (Fig. S1). But, due to little amount of underlying data (n = 3 and n = 2, respectively), these results need to be taken with caution. However, a decreasing trend in well storable varieties (median 5.18% to 4.57%) in comparison to an increasing trend in bad storable varieties (median 3.74% to 4.40%) was detected during storage, so that the level after 13 weeks of storage was similar in all varieties (Fig. S1).

Considering individually measured data over all timepoints, all six varieties with three biological replicates (see Table S2), evidently, fructose, glucose, and invert sugar appeared to be highly positively correlated with each other based on Pearson correlation (Fig. S2). As expected, the combination of potassium and sodium [K + Na] was positively correlated to both individual measurements, potassium [K] and sodium [Na]. On the other hand, sucrose content [°Z] was negatively correlated to sodium, K + Na, fructose, invert sugar, and also glucose, by decreasing order.

In addition, prior to sugar analytics, beets of each variety were assessed regarding root rot (Table S1). Noticeable is, that after 13 weeks of storage (T4), well storable varieties had a higher percentage of healthy beets (V1: 56.7%, V6: 81.1%) than the badly storable varieties (V2: 23.8%, V5: 4.8%).

### Beet root anatomy and lignin quantitation

**Fig. 2:**
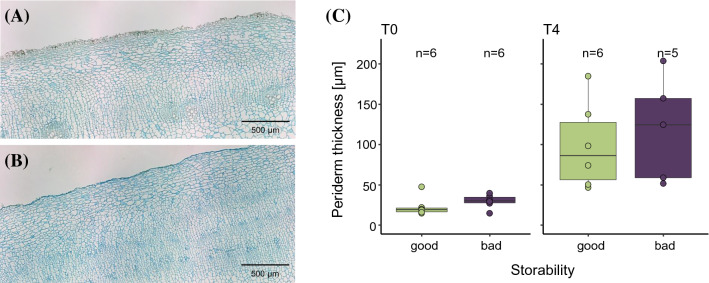
Periderm thickness. Cross section of an individual (62210) from the badly storable variety V5 (**a**) compared to an individual (62216) from the well storable V6 (**b**) at T0. Size bar = 500 µm. Periderm thickness increased during storage (**c**), however, the discrepancy of periderm thickness between good and bad storable varieties at T0 was not seen at T4

Periderm thickness increased in all varieties from an average of 26 µm to an average of 108 µm during storage. Regarding storability, especially at T0, the badly storable varieties were equipped with a thicker periderm. V5 had already a well-developed periderm (average 33 µm), whereas V6 showed only a thin epidermis (average 18 µm) at T0 (Fig. 2). Also, the number of parenchyma cells had the trend to increase 1.5 times during storage in all varieties. Before storage (T0), the cell number was 1.5 times higher in well storable varieties, however, after 13 weeks of storage, this discrepancy between well and badly storable varieties was not visible anymore (Fig. S1 and Table S3). In contrast, the average parenchyma cell area decreased slightly during storage in all varieties. Parenchyma cell area before storage was lower in well storable varieties (median: 2940 µm^2^) compared to the badly storable ones (median: 4429 µm^2^, Fig. S1 and Table S3). After 13 weeks of storage the difference in cell area between well and badly storable varieties (average difference: 723 µm^2^) was not that pronounced as before storage (average difference: 2468 µm^2^). The number of cambial rings did not change during storage, however, a difference between well and badly storable varieties was seen, whereby the former had generally a higher number than the latter (at both timepoints, T0 and T4; Fig. S1 and Table S3).

The lignin content, measured as percent of cell wall material, decreased during storage from an average of 3.3% at T0 to 1.9% at T4 (Fig. S1 and Table S3). There was a trend that the decrease was higher in the badly storable varieties (average of − 1.89%) than in the well storable ones (average of − 0.89%). In the earlier timepoints (T0, T2) lignin content was higher in the badly storable varieties than in the well storable varieties. At T4, the lignin content was equalized between well and badly storable varieties (Fig. S1 and Table S3).

### Phenotypic factors involved in storability

**Fig. 3:**
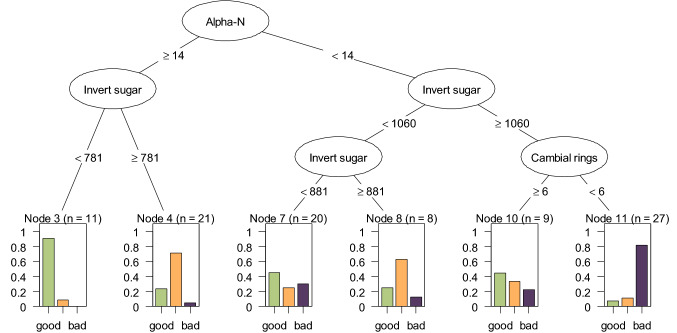
Regression tree analysis. The tree shows the most influencing parameters for storability (out of 11 anatomy and standard analyte parameters tested). The bar plots indicate the percentage distribution of samples belonging to each of the storability classes that fall in that specific tree node. The number in brackets provides the number of all samples in that node. The regression splits into two main branches with one separating good and moderate storability, while the other mainly contains moderate and bad storability observations. Overall, five splits occur which relate to the most relevant variables for differentiating between the storability types: Alpha-amino N, invert sugar content, and the number of cambial rings

To test which factors besides gene expression could be useful for the identification of a good versus a bad storable variety, a regression tree analysis was performed. The main distinctive factors that were identified were alpha-amino N, invert sugar and the number of cambial rings (Fig. 3). In detail, the regression splits into two main branches according to alpha-amino N being less or equal the determined cut-off of 14 mmol/kg to differentiate the majority of the good storable and some moderately storable varieties from the bad and moderate ones. Further down in the tree, these two main branches split again according to the invert sugar content. In the leftmost and central branch, the lower content of invert sugar clearly separates good storability from moderate to bad one. An invert sugar content of less than 781 mg/kg or 881 mg/kg, respectively, is more often associated with good storability. In the rightmost branch with observations with an invert sugar content of at least 1060 mg/kg an additional split happens according to the number of cambial rings which clearly separates the bad storable from the good and moderate storable ones. Having five or fewer cambial rings is clearly associated with bad storability, whereas more than five cambial rings indicate a good storability in high invert sugar samples.

### Transcriptomics

#### Quality control and pre-processing of raw reads

On average, 28 million reads per sample could be obtained. In general, raw reads showed a high base quality, but there was a small amount of N bases and in around 3% of reads Illumina adapters were detectable. After filtering these adapters and low-quality bases as well as cutting off the edges, on average 92% high quality reads with 83% of the bases were obtained for further analysis (Table S2—preprocessing and mapping).

#### Mapping and abundance estimation

The average overall alignment rate over all samples to the reference genome (RefBeet-1.2.2) was 94.6% (~ 25 million reads per sample). On average, 90.3% of these paired-end reads mapped uniquely at least once (Table S2—preprocessing and mapping). As expected, 85.0% mapped in exonic regions, while additionally 9.0% mapped intronic and 6.1% intergenic. The expected counts calculated with featureCounts for each individual sample is presented in form of a PCA plot. The data showed a clear separation into two clusters, whereby one cluster contained only seven samples, six of them from later timepoints (T3 and T4) of badly storable varieties (Fig. S3a). When analyzing each timepoint separately, PCA plots indicated a clear separation of each variety, while at later timepoints individual variation increased. Some outlier samples were observed at T2, T3, and T4 (ID: 62244, 62258, 62277, 62287) and were removed in downstream analyses to increase specificity (Fig. S3b).

#### Pairwise DEG analysis between each variety

A pairwise DEG analysis for each pair of varieties at each timepoint gave a first impression on expressional differences between the varieties and how these differences changed with storage time. On average, an increase of DEGs could be observed with later timepoints (T0 average: 609, T4 average: 1544 DEGs). Further, a high amount of DEGs were seen between the badly storable varieties (V2, V5) compared to better storable varieties at longer storage times (e.g. 2885 DEGs between V2 and V6, and 2883 DEGs between V5 and V6 at T4), while fewest differences were seen between V1 and V4 (Table 1).

**Table 1:**
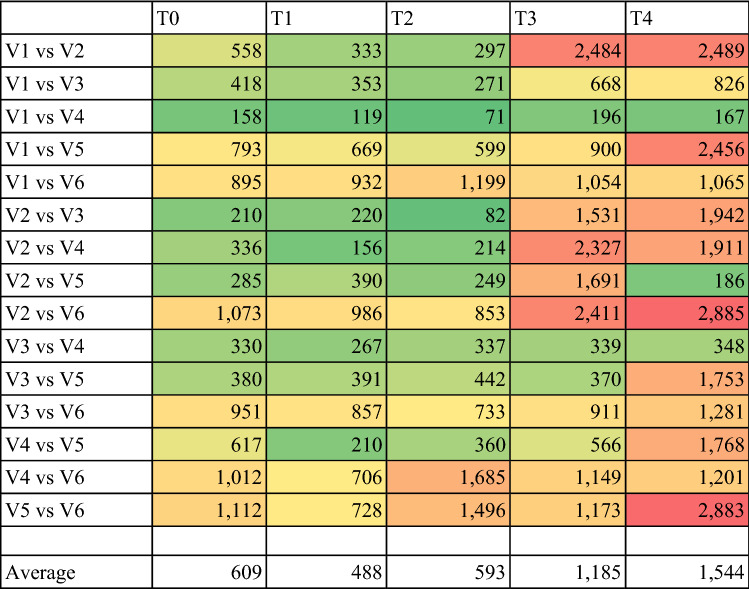
Number of differentially expressed genes (DEGs) per pairwise comparison with a color gradient from green over yellow to red reflecting the range from 71 up to 2885 DEGs, respectively

#### Common changes during storage time

**Fig. 4:**
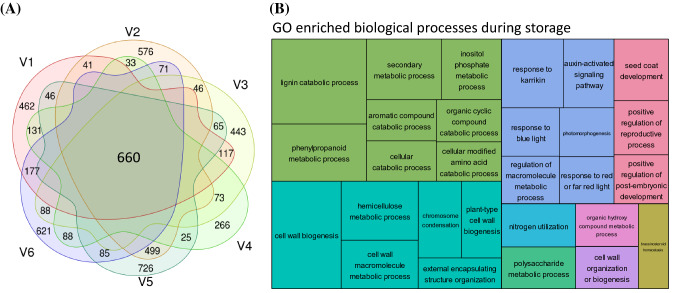
Common changes during storage. Venn diagram showing 660 common significantly differentially expressed genes during storage (**a**). GO enrichment of these genes visualized with REVIGO (**b**)

A pairwise DEG analysis at 13 weeks of storage (T4) in comparison to harvest (T0) was performed for each variety to elucidate transcriptional changes along the time axis. The amount of DEGs for each variety varied between 2754 and 3863. Common significantly differentially expressed genes (Fig. 4a) were declared as the intersection of DEGs of all varieties leading to 257 genes up- and 403 downregulated genes in varieties at T4. The visualization of the GO enrichment analysis for these genes showed the most significant changes during storage in the categories of lignin catabolism or phenylpropanoid metabolism related terms as well as cell wall biogenesis related terms including chromosome condensation (Fig. 4b). A detailed list of all up- and downregulated genes can be found in Table S4. Investigations among DEGs showed e.g. seven xyloglucan endotransglucosylases as well as seven laccases to be downregulated that suggest that sugar beet reduces cell expansion and cell wall structure/integrity during storage (Van Sandt et al. 2007; Wang et al. 2015b; Ranocha et al. 2002). On the other hand, three condensin genes, necessary for chromosome segregation, were upregulated at T4. Within the top 20 upregulated genes (sorted by padj-value) were four cytokinesis related regulators: KEULE, actin-depolymerizing factor, SWR1-complex protein 4, and histone deacetylase 19 (Wu et al. 2013; Maciver and Hussey 2002; Bieluszewski et al. 2015; Tian and Chen 2001), genes that control flowering and light response (APRR5, APRR1, FRIGIDA-like protein 3; Sato et al. 2002; Jiang et al. 2009) and two genes required for resistance to abiotic stresses (zeaxanthin epoxidase, delta(8)-fatty-acid desaturase; Takahashi et al. 2002; Chen et al. 2012). Under the top 20 downregulated genes we found e.g. EXORDIUM-like 3, a gene suggested to act as negative regulatory system for cell division (Farrar et al. 2003; Schröder et al. 2009) as well as two methyltransferases and a phosphatase, that regulates gene expression during development and two genes regulating cell viability (SKIP1, synaptoagmin 2; Hou et al. 2009; Wang et al. 2015a).

#### Changes during storage with regard to the storage potential

**Fig. 5:**
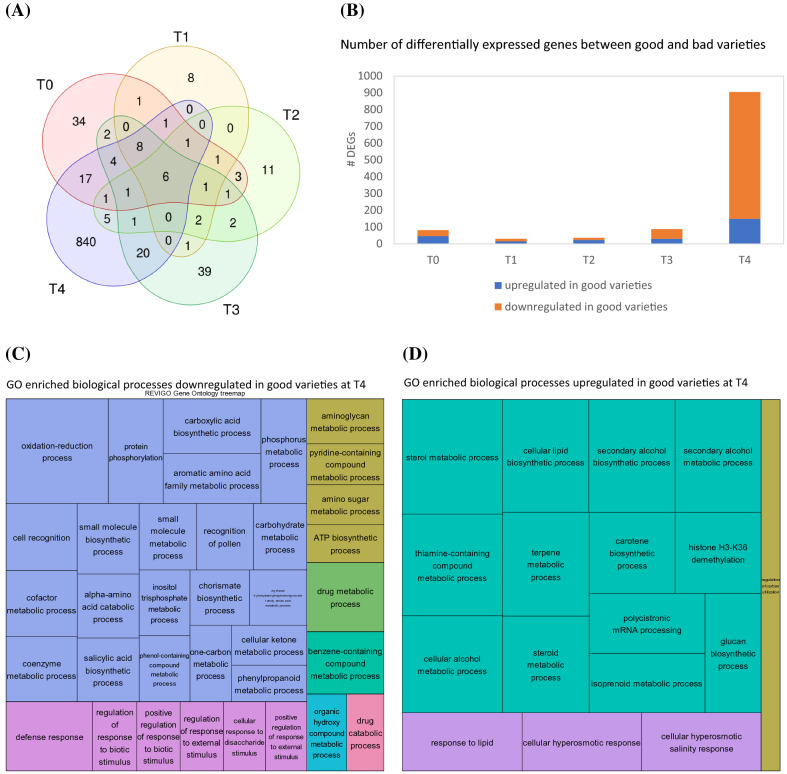
Comparison between good and bad storable varieties. Venn diagram showing the number of differentially expressed genes between well and badly storable varieties at each timepoint (**a**). Histogram of the number of DEGs at each timepoint (**b**). GO enrichment analysis of downregulated genes (**c**) and upregulated genes (**d**) at T4 according biological processes visualized with REVIGO

To identify DEGs between well and badly storable varieties for each timepoint, the intersection of DEGs was computed between all good vs bad varieties (V1 vs V2, V1 vs V5, V6 vs V2 and V6 vs V5) resulting in 82 DEGs at T0, a lower number at T1 and T2 (30 and 36 DEGs, respectively) and up to 905 DEGs at T4 (Fig. 5a, b). In total, 1011 genes were found to be differentially expressed between well and bad storable varieties (Table S5). In general, genes of badly storable varieties were more likely to be higher expressed in comparison to good ones than vice versa. Among the 1011, only six genes were identified to be differentially expressed between well and badly storable varieties in all the timepoints, two of them with a functional annotation, a F-box protein At5g03970-like gene and the organic cation/carnitine transporter 2 with the *Arabidopsis* homolog OCT5.

When focusing on T0 (harvest), already before the storage trial 82 genes could be identified discriminating good from badly storable varieties (Table S5). Noticeable here was the upregulation of two genes responsible for cell divisions (protein TORNADO 2, Kinetochore protein NUF2 homolog; Cnops et al. 2000; Shin et al. 2018) and two downregulated genes involved in cell expansion (Profilin-1, Rhamnogalacturonate lyase family protein; Ramachandran et al. 2000; Ponniah et al. 2017) in the well storable varieties compared to the badly storable varieties. Additionally, we found genes known to play a role in response to abiotic stresses including cell wall modifications e.g. two glycosyltransferases, glutathione S-transferase (Dmitriev et al. 2016), and putative beta glucosidase 41 (Ketudat Cairns and Esen 2010; Le Gall et al. 2015) but also defense related genes against fungi e.g. AX1 (Kragh et al. 1995) and LYM2 (Shinya et al. 2012), that were higher expressed in badly storable varieties.

In contrast to T0, after the entire storage time of 13 weeks (T4), 905 genes differentially expressed between well and badly storable varieties were identified (Fig. 5b). The well storable ones showed an enrichment of genes belonging to e.g. the biological processes sterol metabolism, glucan biosynthetic process and cellular hyperosmotic response (Fig. 5d). On contrary, the badly storable varieties were enriched with genes belonging to e.g. oxidation–reduction processes including cell recognition and carbohydrate metabolic process as well as regulation of response to biotic stimulus for biological processes (Fig. 5c) and the cellular components plant-type cell wall, membrane and extracellular region but also mitochondrial proton-transporting ATP synthase complex (Fig. S4). Under the top 20 upregulated DEGs (sorted by padj-value) in well storable varieties at T4 we found among genes without annotation two genes involved in starch metabolism (starch synthase 1, Turesson et al. 2014; alpha-glucan phosphorylase, Zeeman et al. 2004) and genes important for oxidative stress and pathogen response, e.g. probable thimet oligopeptidase (Moreau et al. 2013), copper methylamine oxidase (Rea et al. 2002) as well as genes positive regulating cell proliferation (*Arabidopsis* homolog At3g07870, Baute et al. 2017; ELP4, Zhou et al. 2009) and one gene involved in sterol biosynthesis (probable 3-beta-hydroxysteroid-Delta(8),Delta(7)-isomerase). Highly downregulated (top 20, sorted by padj-value) were genes important for phenylpropanoid or flavonoid biosynthesis e.g. phenylalanine ammonia-lyase (Ohl et al. 1990), caffeic acid 3-O-methyltransferase, methylenetetrahydrofolate reductase 1 (Liu et al. 2017), cell wall remodeling genes (basic 7S globulin, Yoshizawa et al. 2011; alpha-glucosidase, Gillmor et al. 2002; dirigent protein 22, Paniagua et al. 2017) and stress response genes (glutathione S-transferase, Gullner et al. 2018; pleiotropic drug resistance protein 1, Nuruzzaman et al. 2014). Considering all DEGs downregulated in well storable varieties at T4 we found many more plant stress and defense related genes including six (endo)chitinase genes (Yerzhebayeva et al. 2018) and nine WRKY transcription factors, some of them suggested to regulate stress responses (WRKY6, WRKY15) and others to regulate plants defense against pathogens (WRKY33, WRKY40, WRKY46; Bakshi and Oelmüller 2014). Further, we found 13 Germin-like proteins (GLP) that are known to have an oxalate oxidase function in sugar beet, which leads to the production of hydrogen peroxides, a second messenger molecule during plant stress responses (de los Reyes and McGrath 2003; Gutsch et al. 2018) and six DIR proteins that are known to be plant stress-induced and may play a role in control over cell wall metabolism and/or production of antibacterial compounds (Paniagua et al. 2017) (Table S5).

According DEGs associated to respiration pathways (KEGG database, Kanehisa et al. 2017) we found eight genes downregulated in well storable varieties after 13 weeks of storage (T4) in glycolysis pathway (path:bvg00010), noticeable here hexokinase-1, a fructose and glucose phosphorylating enzyme (Dai et al. 1995) that regulates plant growth and development independently of its sugar metabolism (Jang et al. 1997; Moore et al. 2003) and an ATP-dependent 6-phosphofructokinase, that is known to have a major role in glycolysis of post-harvest sugar beet (Megguer et al. 2017). Ten genes were differentially expressed in the starch and sucrose pathway (path:bvg00500), including the upregulation of three starch synthase related genes in good varieties. Further, we found in well storable varieties a downregulation of two genes in citrate cycle (path:bvg00020, dihydrolipoyl dehydrogenase, mitochondrial and ATP-citrate synthase alpha chain protein 1) and three genes in the electron transport chain and ATP synthase complex (path:bvg00190, protoheme IX farnesyltransferase, ATP synthase subunit delta and epsilon) confirming a higher energy production in badly storable varieties after 13 weeks of storage.

#### KEGG pathway analysis

KEGG enrichment analysis of significant differentially expressed genes between well and badly storable varieties (see sect. "Changes during storage with regard to the storage potential") at T4 showed among others, an enriched downregulation in good varieties in the biosynthesis of secondary metabolites including flavonoid and phenylpropanoid biosynthesis, which is leading to lignin biosynthesis, as well as in the MAPK signaling pathway. An upregulation in well storable varieties is seen in metabolic pathways in general and specifically in thiamine metabolism (Table S6). Interestingly, all stages of the phenylpropanoid pathway were downregulated in good storable varieties (meaning an upregulation in badly storable varieties) exclusively at T4 (Fig. S5).

### WGCNA

**Fig. 6:**
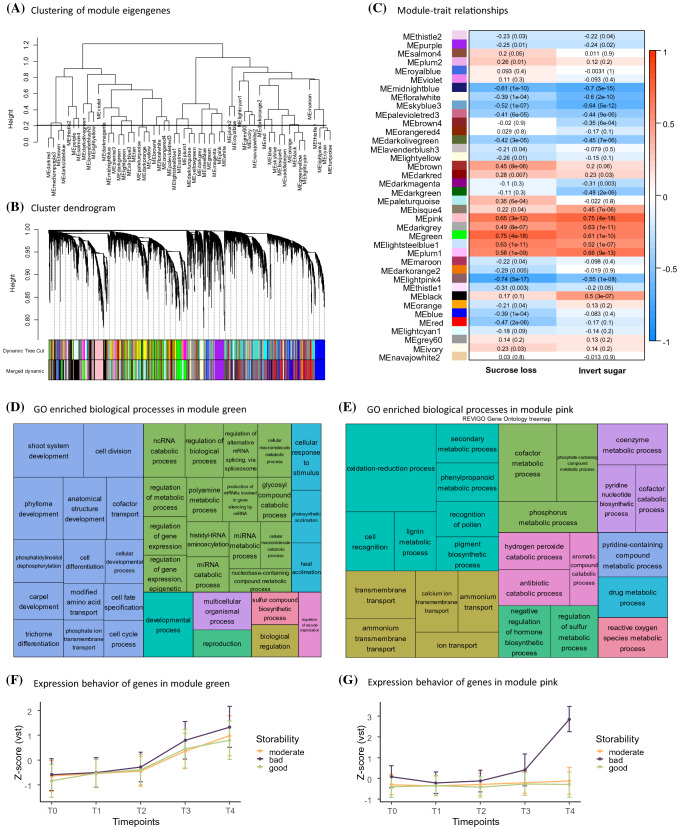
WGCNA. Clustered module eigengenes identified by WGCNA and cutoff for merged modules (**a**). Hierarchical cluster gene tree showing co-expression modules (**b**). The major tree branches form 38 merged modules that are labeled with different colors. Heat map where each cell color shows the correlation of a trait to each WGCNA module eigengene (**c**). GO enrichment analysis of genes in green module (**d**) and genes in module pink (**e**) according biological processes visualized with REVIGO. Expression profile of all genes of the module green correlating to sucrose loss (**f**) and module pink correlating to invert sugar (**g**). The median of variance stabilizing transformed expression values for all samples belonging to one storability group of each gene were determined and the z-score computed. Plots show mean z-score values of all genes and error bars show the standard deviation

A WGCNA analysis was performed including all samples that were also used for DEG analysis (excluding outlier). 19,551 genes were clustered into 26 merged modules (Fig. 6a, b) and highly correlated modules to the most important physiological parameters that define storability, invert sugar and sucrose loss, were further analyzed (Fig. 6c). The highest positive correlation of sucrose loss during storage was seen to the green module (1884 genes, r = 0.75, p = 4e–18) with an enrichment of cell division and development responsible genes (Fig. 6d), and highly negative correlated was the module lightpink4 (3140 genes, r =  − 0.74, p = 5e–17) with an enrichment of e.g. regulation of defense response to bacteria (Fig. S6a) The highest correlation of invert sugar was seen to the module pink (1086 genes, r = 0.75, p = 4e–18) composed of genes showing an enrichment of e.g. secondary metabolism (lignin, phenylpropanoid), coenzyme metabolism or reactive oxygen species metabolic processes (Fig. 6e). Whereas a negative correlation (436 genes, r =  − 0.7, p = 5e–15) was given to the module midnightblue, enriched, among others, by cellular response to abiotic stimulus and nitrogen compound metabolic process (Fig. Sb). Genes in the green module show a general increase of expression in the course of storage time (Fig. 6f) while genes in module pink showed a high increase of expression at T4 in badly storable varieties exclusively (Fig. 6g). Further, hub genes of highly correlated genes were determined, taking into account the intramodular connectivity of WGCNA analysis and additionally the connectivity degree of known protein–protein interactions. Five hub genes could be defined that are associated with sucrose loss and six genes associated with invert sugar. Hub genes for sucrose loss include three genes acting as or with chaperones (dnaJ protein ERDJ2A, nucleotide exchange factor SIL1, transcription elongation factor SPT6 homolog; Ohta and Takaiwa 2014; Behnke et al. 2015; Duina 2011) and a gene involved in glycolysis (triosephosphate isomerase, cytosolic, Dumont et al. 2016). Those for invert sugar include three chalcone synthases and a caffeoyl-CoA O-methyltransferase (Table S7).

### Analysis of unmapped reads

Analysis of unmapped reads showed presence of fungi as well as virus annotated transcripts. Five different viruses (Beet cryptic virus 2, Beet necrotic yellow vein virus, Beet virus Q, *Beta vulgaris* mitovirus 1, Melon chlorotic spot virus) could be detected in at least one individuum. Differences according storability were seen for Beet cryptic virus 2 that is almost double more likely to be detected in badly storable varieties (Fig. S7). In single individuals also a noticeable amount of fungi transcripts (total counts > 10) were detected at later timepoints (T2–T4), that included the genera *Colletotrichum*, *Alternaria*, *Cryptococcus* and *Botrytis* (Table S8).

## Discussion

In this study we performed comparative transcriptomics together with sugar and standard analyte, and anatomical analyses of the roots of six sugar beet varieties in a storage trial. We found factors and mechanisms with a variety-effect regarding storability that were able to discriminate well and badly storable varieties and their pattern during a storage period of 13 weeks.

### Sucrose consumption for combating drought stress and pathogen attacks—common effects during storage

Generally, in all varieties, the SC was decreasing during the entire storage time whereas the amount of invert sugar increased; two well-known effects during storage representing the main challenges of the sugar industry. In addition, we saw an overall increase in parenchyma cell number during storage, which was supported by the expression of genes important for cell division, whereas a prevention of cell expansion (downregulation of seven xyloglucan endotransglucosylases) and reduction of cell wall integrity, likely due to the downregulation of seven laccases, seemed to occur. Laccases are known to be involved in biosynthesis and degradation of lignin (Berthet et al. 2012) but there were no significant differences identified in the overall lignin content after storage (see Lignin quantitation/Fig. S1). The higher expression of laccases at T0 could thus also be explained as wound healing reaction that occurred directly after harvesting (Wang et al. 2015b). Further, an overall increase of periderm thickness was recognized during storage, most likely for a more controlled regulation of water and gas exchange as well as for an improved protection from biotic and abiotic stresses, especially after wounding (Fugate et al. 2016; Campilho et al. 2020). That is supported by the high upregulation of genes (e.g. zeaxanthin epoxidase, delta(8)-fatty-acid desaturase) that are required for resistance to osmotic and drought stress to cope with water stress during storage (Harrison et al. 2006). Since the periderm is made up of suberized cells (Graça 2015), we expected, that important enzymes in the phenylpropanoid pathway  (e.g. phenylalanine ammonia lyase, PAL, and peroxidases, POD) were expressed during storage. However, our study supported previous findings, that periderm synthesis in sugar beet is not related to the activity of PAL and POD (Fugate et al. 2016). But, a gene very likely involved in this process might be glucan endo-1,3-beta-glucosidase 11, a homolog to the plasmodesmata-associated beta-1,3-glucanase in *Arabidopsis*, playing a role in development and defense processes (Levy et al. 2007).

It seems that general sucrose loss during storage in sugar beet goes hand in hand with the change in growth strategy that is characterized by decreased cell expansion and increased cell division (Zhang et al. 2017; Lastdrager et al. 2014) and further periderm development. That a physiological and molecular response to abiotic stress is inevitably taking place during storage has been shown already (e.g. Klotz and Finger 2004; Campbell and Klotz 2007a; Schnepel and Hoffmann 2014; Liebe and Varrelmann 2016). We detected an increased expression of genes related to pathogen defense along the storage axis too, however, a defense response strategy that is common for all six studied varieties could not be seen, likely indicating variety-specific response mechanisms.

### The preconditions for being a well storable variety

The better storable varieties were equipped with a higher SC, a higher number of cambial rings and more parenchyma cells, while the cell size itself was smaller in comparison to badly storable varieties at T0. This could be confirmed with comparative transcriptomics, where two upregulated genes responsible for cell division, protein TORNADO 2 and Kinetochore protein NUF2 homolog (Cnops et al. 2000; Shin et al. 2018), and two downregulated involved in cell expansion, Profilin-1 (Ramachandran et al. 2000) and Rhamnogalacturonate lyase family protein (Ponniah et al. 2017) were identified in the well storable varieties. That beet varieties containing a high SC have more and smaller parenchyma cells was already found in previous anatomical and transcriptomic analysis (Zhang et al. 2017; Slater et al. 2014; Doney et al. 1981). One statement is, that cell enlargement moves the cell further away from the vascular zones, resulting in less sucrose concentration due to declined sink capacity (Draycott 2006). This effect could be shown in a study were the amount of sucrose in sugar beet only correlated with cell volume at the initial state of cell expansion, while it was decreasing after a certain size in contrast to water and non-sucrose compounds (Milford 1973). A similar finding was described for potatoes too, where a higher percentage of smaller cells increased tissue resistance, which could potentially be associated with better storability (Konstankiewicz and Zdunek 2001). Such an effect might influence storability in sugar beet too: the above mentioned Rhamnogalacturonate lyase family protein, that was significantly downregulated in well storable varieties, is involved in the degradation of cell-wall middle lamellae and thus might play a role in the fruit ripening-related softening process reducing fruit firmness and post-harvest life (Molina-Hidalgo et al. 2013).

That well storable varieties showed a thinner periderm together with less lignin content than badly storable ones seemed, at first sight, astonishing; we expected the vice versa scenario. Since lignin is known to be an important barrier for pathogens, we thus hypothesize, that badly storable varieties had the need to establish lignified cell walls already earlier in their development to overcome their susceptibility to pathogen attacks, which might be caused by a significant upregulation of DIR23, a gene involved in lignin biosynthesis (Paniagua et al. 2017), which is significantly downregulated in well storable varieties. In addition, well storable varieties seem to be equipped with a different, obviously more efficient defense system, likely caused by the significant upregulation of a member of the beta glucosidase gene family (BGLU41) hinting towards an immediate chemical defense against pathogens (Morant et al. 2008). In addition, a higher marc content was found to be associated to good storability, supporting previous findings (Schnepel and Hoffmann 2016; Hoffmann et al. 2018), where a higher marc content already before storage was seen. The hypothesis, whether a high marc content is correlated to a smaller cell size (Drath et al. 1984; Hoffmann et al. 2018) can likely be now confirmed by this study. However, care needs to be taken with these results since data density at T0 was little (Fig. S1), making a follow-up study necessary.

In summary, already at time of harvest (T0), prior to storage, well and badly storable varieties can be discriminated in their response to abiotic stresses, amongst others, by showing a different expression of cell wall modification genes and also a different reaction to pathogen attack.

### Bad storability goes hand in hand with increased stress response and pathogen defense

The upregulation of the starch metabolism in well storable varieties during storage seen in our study seems very interesting as it is known that sugar beet stores energy only in form of sucrose instead of starch, despite an expression of starch biosynthesis genes (Turesson et al. 2014). However, a gene that appeared highly upregulated in well storable varieties was an alpha-glucan phosphorylase, which is part of the starch metabolic process that is known to endure water stress deficit but does not alter starch content in *Arabidopsis* (Zeeman et al. 2004). A better cope with the hyperosmotic cell state most likely due to the water stress during storage can be further seen in well storable varieties as well as an increase in oxidative stress response. Interestingly, the mitochondrial dihydrolipoyl dehydrogenase was downregulated in well storable varieties, indicating a tight regulation of cell respiration during storage between well and badly storable varieties (Timm et al. 2015). Furthermore, the downregulation of genes in the electron transport chain in well storable varieties and the enrichment of carbohydrate metabolism of downregulated DEGs confirmed a higher energy production and consumption in badly storable varieties during storage.

A further factor that might have a major impact on a variety’s storability was alpha-amino N content, whereby the higher the better for a good storability (≥ 14, see Regression tree analysis). It is long known, that plants are able to respond rapidly to stressors by increasing the concentration of compatible solutes involved in osmoregulation, such as nitrogen-rich compounds, as already described for potato and sugar beet (Levy 1983; Gzik 1996). It is likely, that good storable varieties accumulate alpha-amino N to protect the beet root against osmotic stress that occurred during storage. However, a high accumulation of this compound is of disadvantage for sugar beet processing, since it interferes with the sucrose extraction.

Correlation of invert sugar content to the expression data of all studied varieties and timepoints via a gene co-expression network analysis (WGCNA) and further functional analysis showed, that most correlated genes were associated to defense and stress response. That is mainly caused by the high upregulation of genes of badly storable varieties at T4. The expression of genes highly correlated to sucrose loss is increasing during storage, slightly faster in badly storable varieties. These genes were identified to be involved in the regulation of cell growth and development. The enrichment of genes in modules highly negatively correlated to invert sugar and sucrose loss showed, that not all stress and defense related genes are higher expressed in badly varieties at 13 weeks of storage; a significant amount is higher expressed in well storable varieties that might include important genes for abiotic stress (e.g. water deficit) and pathogen resistance. For example, we found two genes required for resistance to the plant pathogen *Alternaria brassicicola*, a homolog to *Arabidopsis* MAPKKK5 and coronatine-insensitive protein 1 (Yamada et al. 2016; van Wees et al. 2003), a disease resistance protein RPS2 required for resistance to the bacterium *Pseudomonas syringae* (Mackey et al. 2003), glucosidase 2 subunit beta that determines the perception of the bacterial elongation factor Tu (Lu et al. 2009), or the ethylene-insensitive protein 2, that is required for salt tolerance (Lei et al. 2011).

That defense mechanisms against pathogens were activated predominantly in badly storable varieties was supported by the analysis of unmapped reads, that gave a first hint of viral and fungal infestations in relation to storability. Especially three of the discovered fungi genera (*Alternaria*, *Botrytis* and *Colletotrichum*) in the unmapped reads are known to start plant cell-wall mediated immunity response (Bacete et al. 2018). Further, we found the presence of beet cryptic virus 2 (BCV2) almost twice as likely in badly storable varieties than in good ones, while it is known that BCV infection can reduce root and sucrose yield up to 20% (Xie et al. 1994). We are aware that taking the unmapped reads can provide us just with a glimpse on the microbial community setup with constraints towards fungal and viral transcripts. Thus, a further analysis is necessary to investigate differences in microbial communities between well and badly storable varieties.

### Hub genes related to storability

With WGCNA we could further define several hub genes that correlate to sugar beet storability and could act as putative marker genes. Among six hub genes found to be highly correlated to invert sugar were three chalcone synthases, key enzymes of the flavonoid biosynthesis pathway that are also known to be induced under abiotic and biotic stresses and play a major role in plant resistance (Dao et al. 2011), supporting our hypothesis of an increased stress response in badly storable varieties. Additionally, a caffeoyl-CoA O-methyltransferase was found, also involved in flavonoid biosynthesis but especially in lignification (Fellenberg et al. 2012), likely hinting towards a stress-induced biosynthesis of phenylpropanoids in badly storable varieties, whereby a higher lignification could not be confirmed in our study. Among hub genes highly correlated to sucrose loss we found three that are chaperones or act with chaperones. Two are involved in the HSP70 system (dnaJ protein ERDJ2A, nucleotide exchange factor SIL1; Ohta and Takaiwa 2014; Behnke et al. 2015), that helps proteins to reach their native conformation or regain function after misfolding due to various stress conditions (Sharma and Masison 2009). A further hub gene for sucrose loss is cytosolic triosephosphate isomerase, thought to modulate ROS production as a resistance mechanism, already shown against *Xanthomonas oryzae* in rice (Liu et al. 2018).

## Conclusion

Based on comparative transcriptomics and integration of beet root anatomy, sugar and standard analyte data of six varieties we identified key factors influencing sugar beet storability (defined as sucrose loss and invert sugar accumulation during storage). Besides common effects during storage, we narrowed down genotypic differences prior to storage and during the storage trial of 13 weeks. Varieties that were equipped with a higher number of parenchyma cells and cambial rings as well as a thinner periderm prior to storage showed a better storability behavior. In addition, the downregulation of genes involved in fruit ripening-related softening processes seemed to be a potential precondition for good storability as well as the upregulation of a specific, obviously more efficient pathogen defense system. After 13 weeks of storage, however, well storable varieties seemed to better cope with the hyperosmotic cell state, showed a downregulation of cell respiration and carbohydrate metabolism, as well as less defense and stress response. Interestingly, a higher alpha-amino N content in well storable varieties was detected, hinting towards an osmoprotective function during storage. In the end, the characterization of hub genes that correlate to sugar beet storability could additionally act as putative marker genes.

## Electronic supplementary material

Below is the link to the electronic supplementary material.

Supplementary file1 (XLSX 17 kb) Table S1 Sack-wise measurements including the average weight per sack and variety at T0, and at the sampling timepoints, and the calculated weight loss per sack [kg]. Further, sucrose content per sack and variety at T0, and at the sampling timepoints, and the calculated absolute sucrose loss as well as the relative sucrose loss, the latter normalized by the overall weight loss and depicted in percent. In addition, phenotyping results of root rot at T4 is given in percent

Supplementary file2 (XLSX 52 kb) Table S2 Metadata of each individual beet root of each variety and timepoint including storability information, sugar and standard analyte measurements, sucrose loss in percent, and marc content (percent) are given in sheet 1 (sugar analytics). Read statistics of each individual sample from raw reads to the overall alignment rate to percent of mapped reads is depicted in sheet 2 (preprocessing and mapping).

Supplementary file3 (XLSX 29 kb) Table S3 Anatomical parameters for each individual sample at T0 and T4, including periderm thickness [µm²], absolute number of cambial rings at the cross section, number of parenchyma cells in a defined section, and total root weight [g]. In addition, lignin quantification (in % of cell wall material) was done at T0, T2, and T4

Supplementary file4 (XLSX 114 kb) Table S4 Information of all downregulated (Sheet 1) and upregulated (Sheet 2) DEGs after 13 weeks of storage (T4) compared to prior to harvest (T0)

Supplementary file5 (XLSX 200 kb) Table S5 All DEG genes between well and badly storable varieties at each timepoint are provided in sheet 1 (overview). ↑ indicates that the gene was significantly upregulated, ↓ indicates that the gene was significantly downregulated, and - indicates that the gene was not differentially expressed. Additional sheets: detailed information of DEG genes at T0 and T4, padj-value sorted

Supplementary file6 (XLSX 19 kb) Table S6 Enriched KEGG pathways of DEGs of well versus bad storable varieties at T4. Pathways are grouped by KEGG hierarchy levels

Supplementary file7 (XLSX 1164 kb) Table S7 Correlated genes to sucrose loss (sheet 1) and to invert sugar (sheet 2) in WGCNA modules: All genes of the WGCNA modules green and lightpink4 (correlated to sucrose loss), and the modules pink and midnightblue (highly correlated to invert sugar) are listed. Column variables are explained in Methods section. Sorting according to corresponding gene significance (GS1). Hub genes are marked in yellow and are shown at the top

Supplementary file8 (XLSX 12 kb) Table S8 Sugar beet individuals that have more than ten assembled genes of unmapped reads annotated to fungi genera

Supplementary file9 (PPTX 173 kb) Fig. S1 Anatomical analyses of well (variety 1 and 6, green) and badly storable (variety 2 and 5, violet) sugar beet varieties: Marc content [%], parenchyma cell area [µm²], parenchyma cell number, total number of cambial rings, and lignin concentration as percent of cell wall material. Analysis was done with beets sampled after harvest (T0) and after 13 weeks of storage (T4), additionally after two weeks of storage (T2) for the lignin content. Per variety and sampling point at maximum three biological replicates were analyzed, represented by the dots in the boxplots

Supplementary file10 (PNG 12 kb) Fig. S2 Pearson correlation analysis of sugars, standard analytes, and marc content over all samples and timepoints. Being obvious, positively correlated were glucose and fructose with invert sugar, as well as potassium and sodium with K+Na. Sucrose content was negatively correlated with sodium, K+Na, fructose, glucose, and invert sugar

Supplementary file11 (PPTX 72 kb) Fig. S3 PCA plots of variance stabilizing transformed expression values of top 500 genes with highest variance across samples (a). PCA plots separated by storage timepoints. Outlier samples that are removed for further analysis are indicated by an arrow (b)

Supplementary file12 (PPTX 37 kb) Fig. S4 GO enrichment analysis of downregulated genes at T4 according cellular components visualized with REVIGO

Supplementary file13 (PNG 33 kb) Fig. S5 Significantly up-/downregulation of genes in well storable vs badly storable varieties in the phenylpropanoid pathway. Each KEGG enzyme (EC number) is colored according the sum of logFC for corresponding genes at timepoint 0 (left), 2 (middle) and 4 (right). If a KEGG enzyme is shown in blue, corresponding genes were upregulated in good varieties, if orange, genes were downregulated in good varieties, and white, if genes were not significantly differentially expressed or if no genes were assigned to that EC number yet

Supplementary file14 (PPTX 68 kb) Fig. S6 GO enrichment analysis of genes in lightpink4 module (a) and genes in midnightblue (b) module according biological processes visualized with REVIGO

Supplementary file15 (PPTX 42 kb) Fig. S7 Number of individuals (in percent) that had at least one assembled gene of unmapped reads annotated to virus taxa, separated by storability behavior from good (green) over orange (moderate) to violet (bad)Supplementary table captions

## Data Availability

Pre-processed raw sequence reads of all 96 samples are available on NCBI SRA database, accession number PRJNA610534.
