# Supplementary material for: Integrative transcriptomics reveals genotypic impact on sugar beet storability
Source: Plant Mol Biol. 2020 Aug 4;104(4):359–78. doi: 10.1007/s11103-020-01041-8 (PMC7593311; doi:10.1007/s11103-020-01041-8)
Supplement: Supplementary file 9 — Supplementary file9 (PPTX 173 kb) Fig. S1 Anatomical analyses of well (variety 1 and 6, green) and badly storable (variety 2 and 5, violet) sugar beet varieties: Marc content [%], parenchyma cell area [µm²], parenchyma cell number, total number of cambial rings, and lignin concentration as percent of cell wall material. Analysis was done with beets sampled after harvest (T0) and after 13 weeks of storage (T4), additionally after two weeks of storage (T2) for the lignin content. Per variety and sampling point at maximum three biological replicates were analyzed, represented by the dots in the boxplots [file 11103_2020_1041_MOESM9_ESM.pptx]

## Slide 1
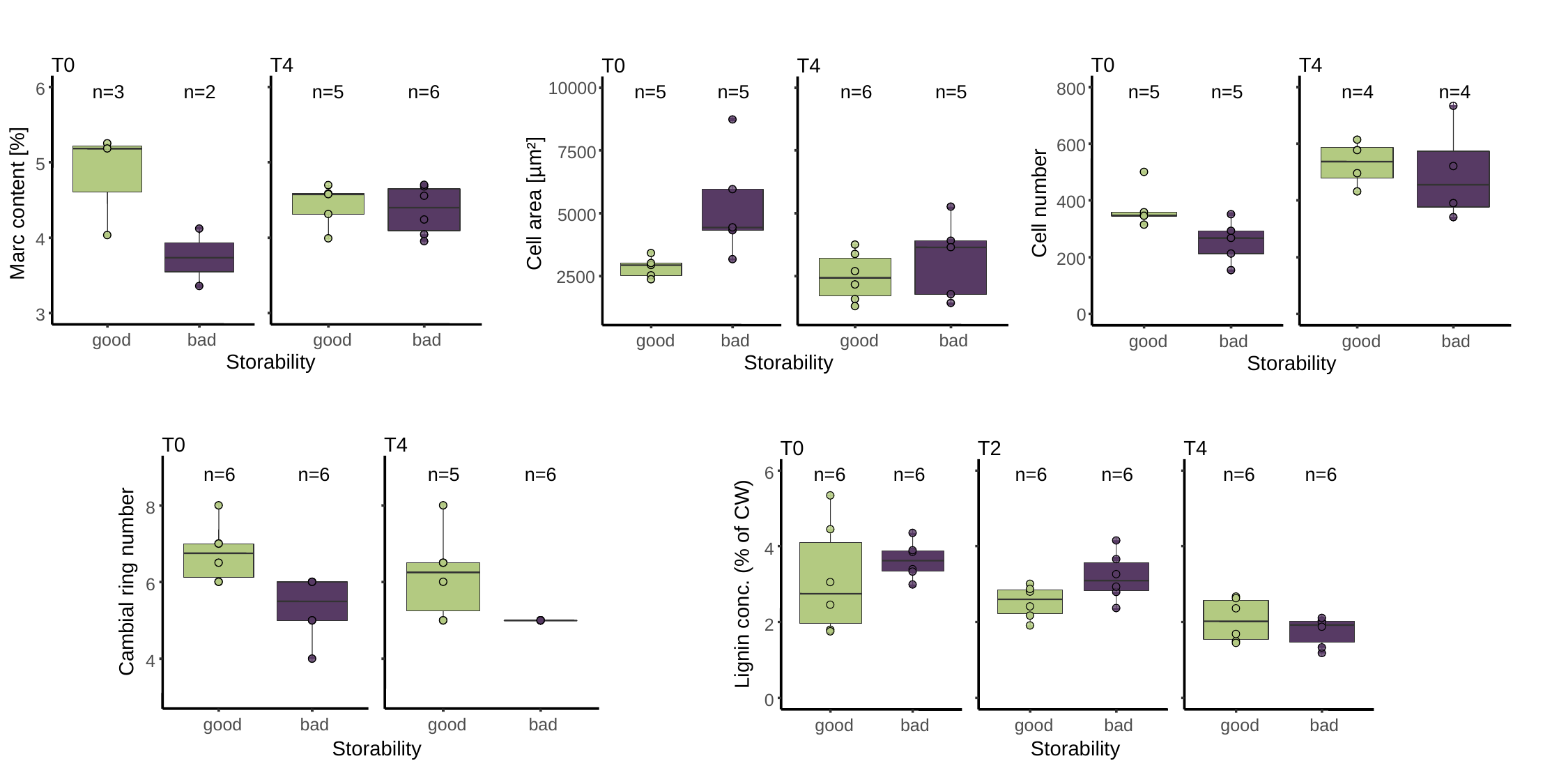

T0
T4
T0
T4
T0
T4
10000
6
800
n=3
n=2
n=5
n=6
n=5
n=5
n=6
n=5
n=5
n=5
n=4
n=4
600
7500
5
400
Cell number
Cell area [µm²]
Marc content [%]
5000
4
200
2500
3
0
good
bad
good
bad
good
bad
good
bad
good
bad
good
bad
Storability
Storability
Storability
T0
T4
T0
T2
T4
6
n=6
n=6
n=6
n=6
n=5
n=6
n=6
n=6
n=6
n=6
8
4
Cambial ring number
Lignin conc. (% of CW)
6
2
4
0
good
bad
good
bad
good
bad
good
bad
good
bad
Storability
Storability
