# Supplementary figures and images for: Integrative transcriptomics reveals genotypic impact on sugar beet storability
Source: Plant Mol Biol. 2020 Aug 4;104(4):359–78. doi: 10.1007/s11103-020-01041-8 (PMC7593311; doi:10.1007/s11103-020-01041-8)

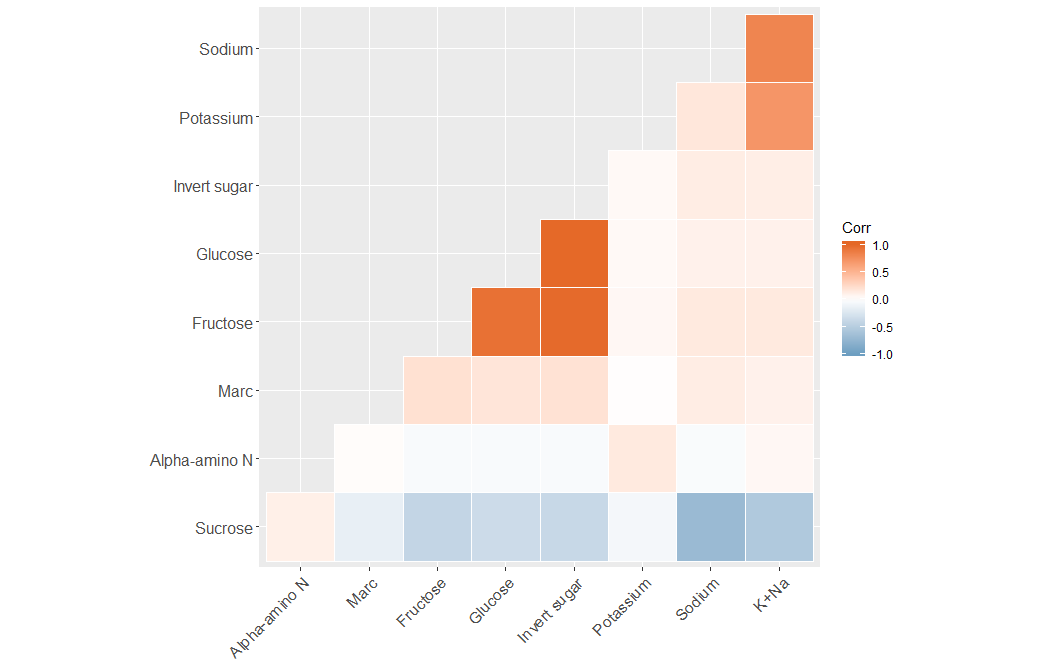

Supplement: Supplementary file 10 — Supplementary file10 (PNG 12 kb) Fig. S2 Pearson correlation analysis of sugars, standard analytes, and marc content over all samples and timepoints. Being obvious, positively correlated were glucose and fructose with invert sugar, as well as potassium and sodium with K+Na. Sucrose content was negatively correlated with sodium, K+Na, fructose, glucose, and invert sugar [file 11103_2020_1041_MOESM10_ESM.png]

## Slide 1
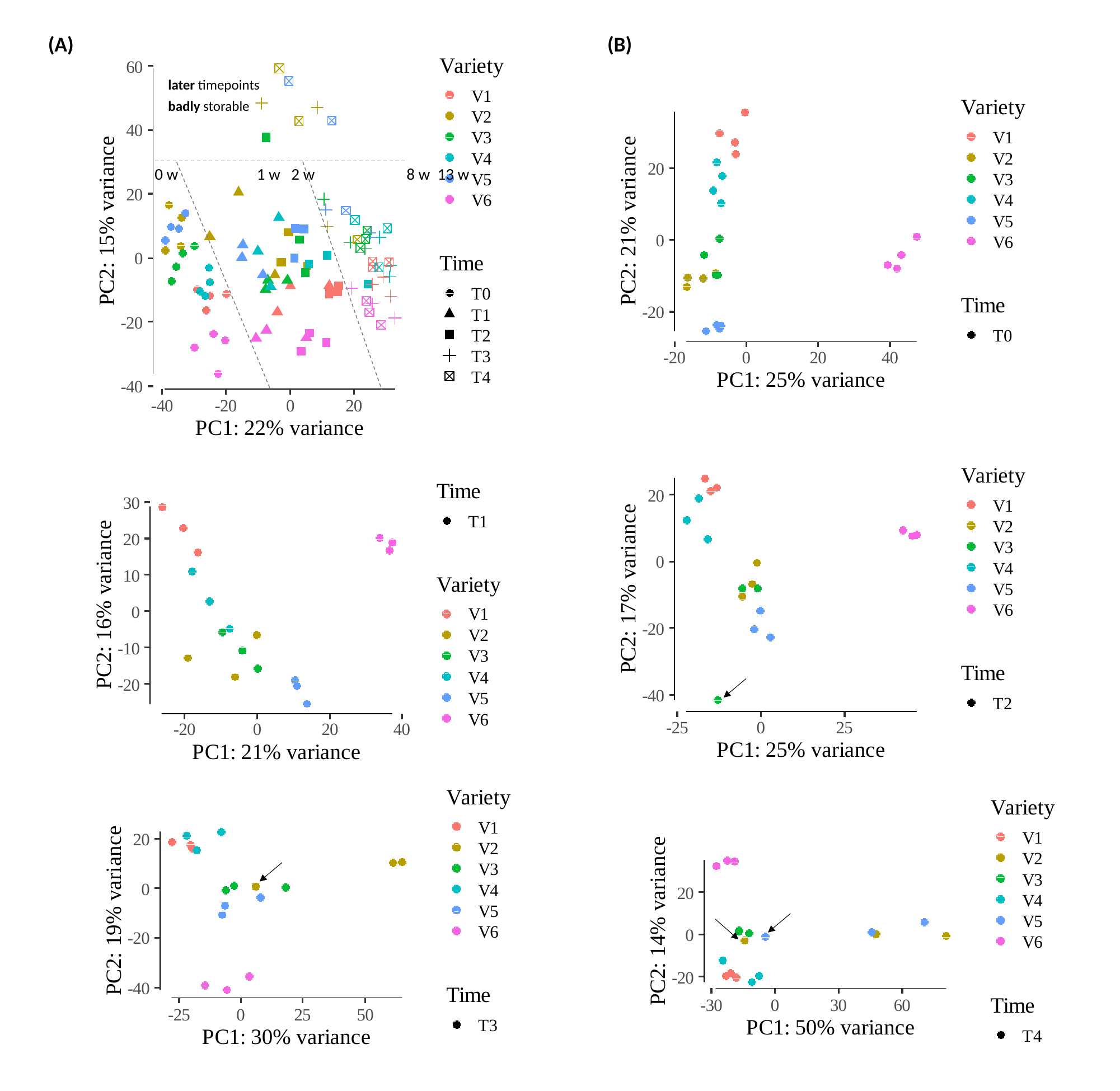

(A)
(B)
later timepoints
badly storable
0 w	 1 w 2 w 	8 w 13 w

Supplement: Supplementary file 11 — Supplementary file11 (PPTX 72 kb) Fig. S3 PCA plots of variance stabilizing transformed expression values of top 500 genes with highest variance across samples (a). PCA plots separated by storage timepoints. Outlier samples that are removed for further analysis are indicated by an arrow (b) [file 11103_2020_1041_MOESM11_ESM.pptx]

## Slide 1
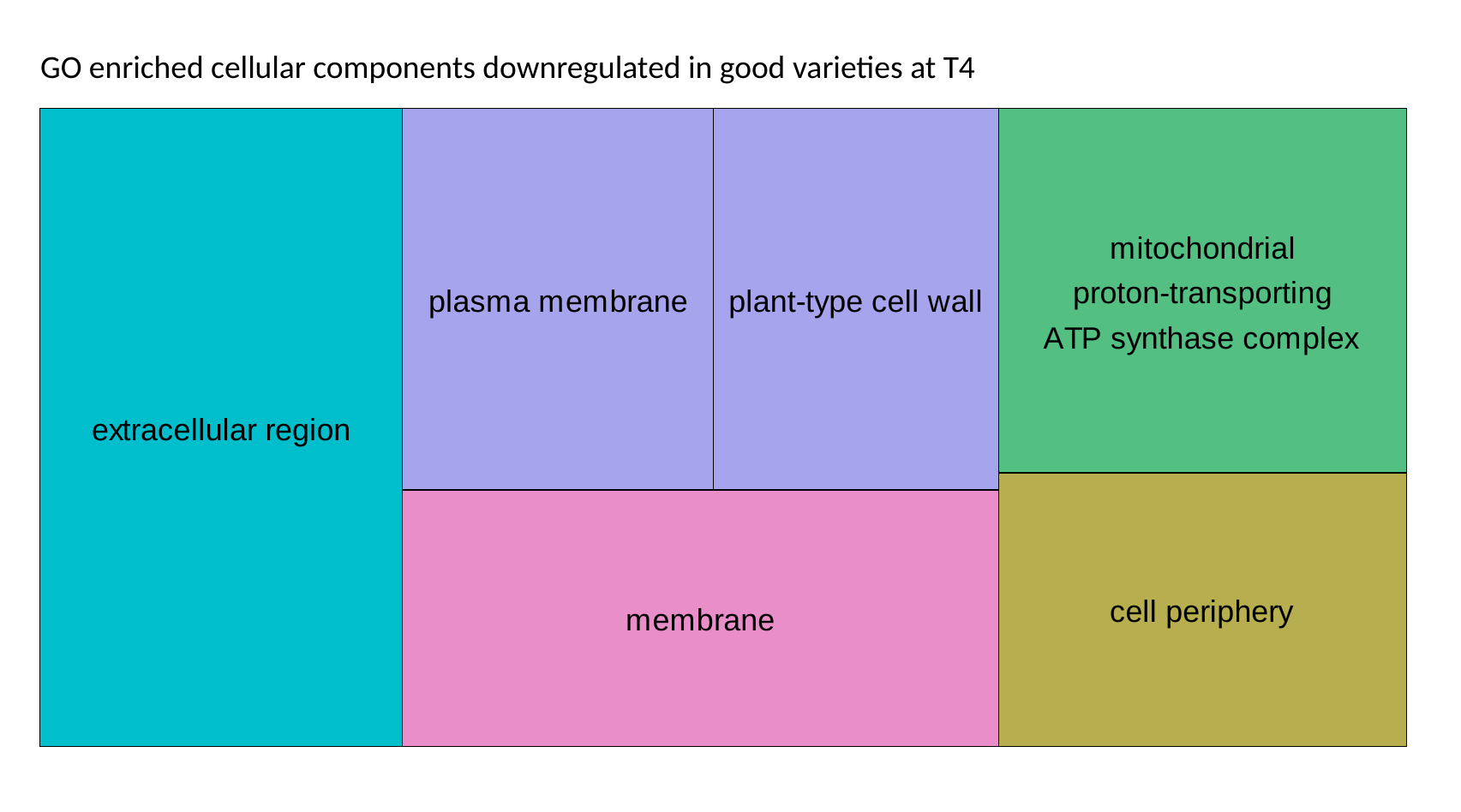

GO enriched cellular components downregulated in good varieties at T4

Supplement: Supplementary file 12 — Supplementary file12 (PPTX 37 kb) Fig. S4 GO enrichment analysis of downregulated genes at T4 according cellular components visualized with REVIGO [file 11103_2020_1041_MOESM12_ESM.pptx]

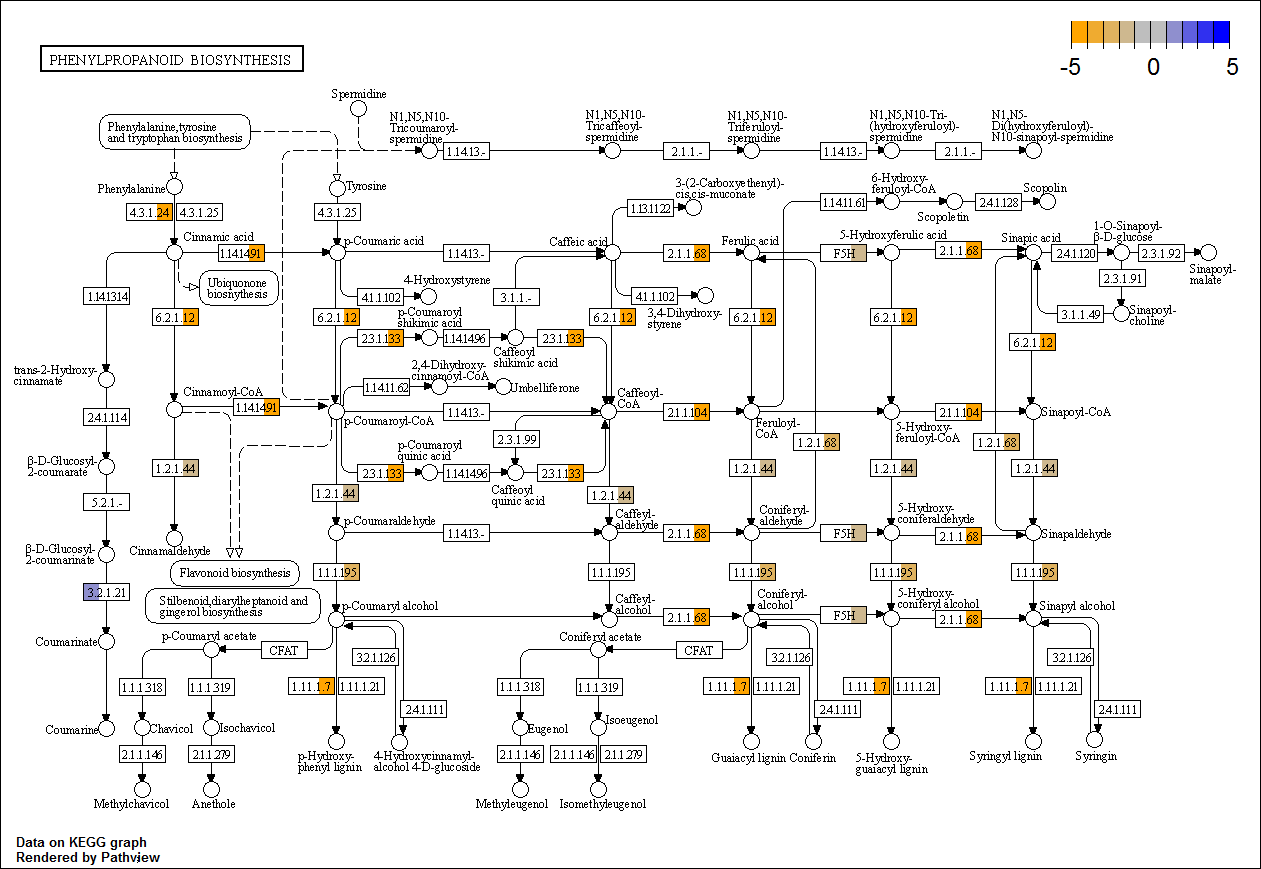

Supplement: Supplementary file 13 — Supplementary file13 (PNG 33 kb) Fig. S5 Significantly up-/downregulation of genes in well storable vs badly storable varieties in the phenylpropanoid pathway. Each KEGG enzyme (EC number) is colored according the sum of logFC for corresponding genes at timepoint 0 (left), 2 (middle) and 4 (right). If a KEGG enzyme is shown in blue, corresponding genes were upregulated in good varieties, if orange, genes were downregulated in good varieties, and white, if genes were not significantly differentially expressed or if no genes were assigned to that EC number yet [file 11103_2020_1041_MOESM13_ESM.png]
