# Supplementary material for: Integrative transcriptomics reveals genotypic impact on sugar beet storability
Source: Plant Mol Biol. 2020 Aug 4;104(4):359–78. doi: 10.1007/s11103-020-01041-8 (PMC7593311; doi:10.1007/s11103-020-01041-8)
Supplement: Supplementary file 14 — Supplementary file14 (PPTX 68 kb) Fig. S6 GO enrichment analysis of genes in lightpink4 module (a) and genes in midnightblue (b) module according biological processes visualized with REVIGO [file 11103_2020_1041_MOESM14_ESM.pptx]

## Slide 1
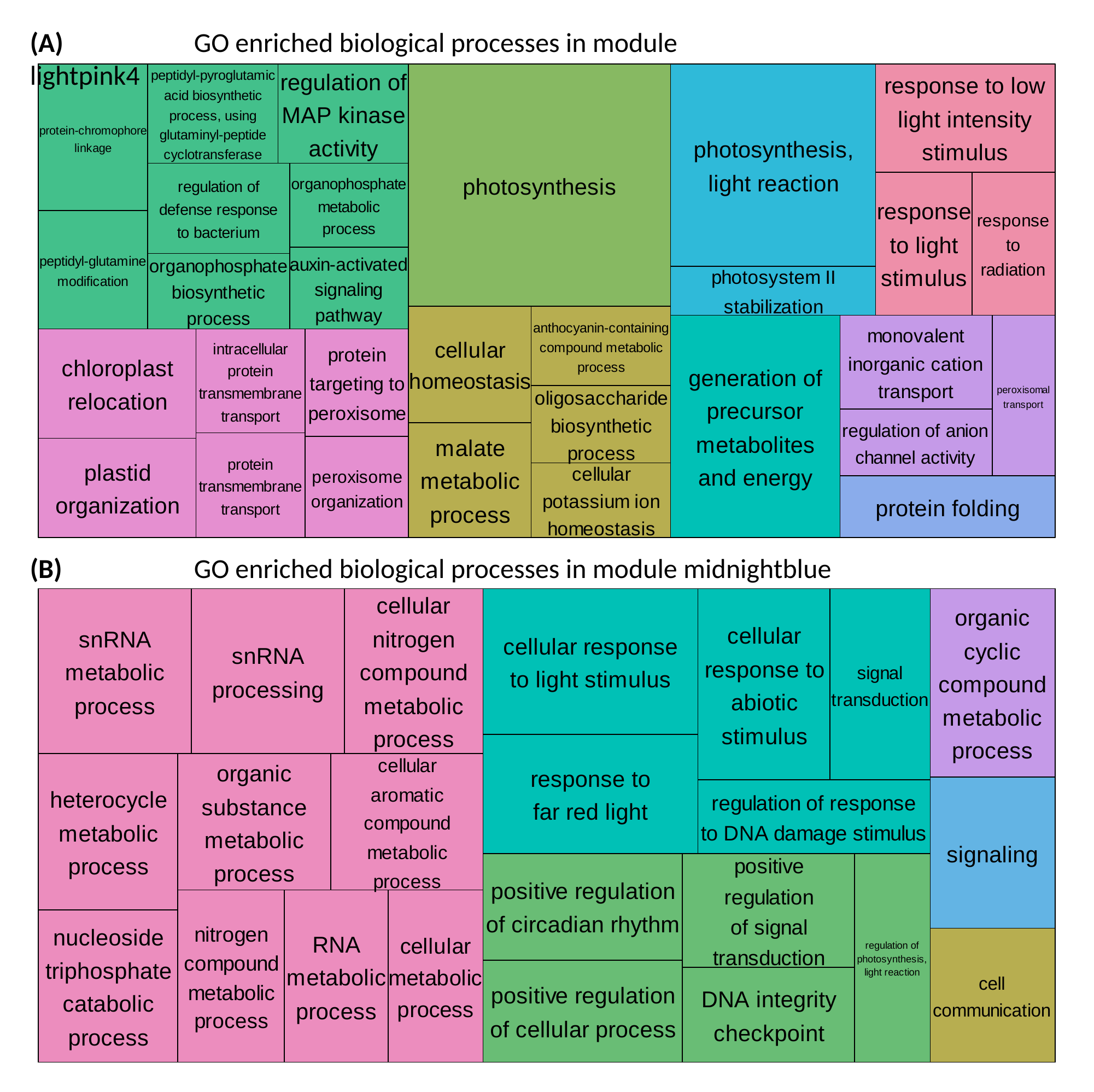

(A)		GO enriched biological processes in module lightpink4
(B)		GO enriched biological processes in module midnightblue
