# Supplementary material for: Integrative transcriptomics reveals genotypic impact on sugar beet storability
Source: Plant Mol Biol. 2020 Aug 4;104(4):359–78. doi: 10.1007/s11103-020-01041-8 (PMC7593311; doi:10.1007/s11103-020-01041-8)
Supplement: Supplementary file 15 — Supplementary file15 (PPTX 42 kb) Fig. S7 Number of individuals (in percent) that had at least one assembled gene of unmapped reads annotated to virus taxa, separated by storability behavior from good (green) over orange (moderate) to violet (bad)Supplementary table captions [file 11103_2020_1041_MOESM15_ESM.pptx]

## Slide 1
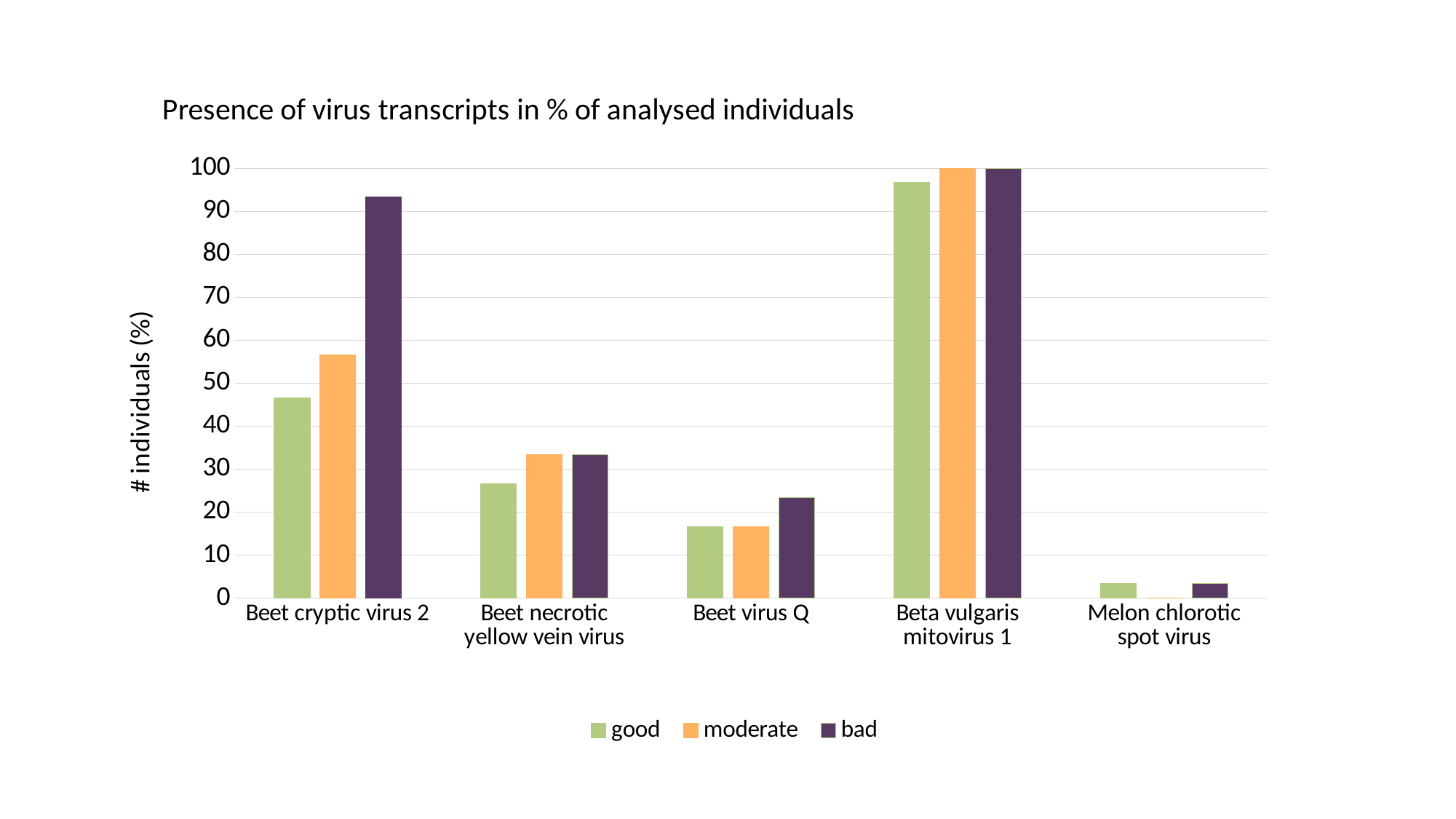

### Chart: Presence of virus transcripts in % of analysed individuals
| Category | good | moderate | bad |
|---|---|---|---|
| Beet cryptic virus 2 | 46.66666666666667 | 56.66666666666667 | 93.33333333333334 |
| Beet necrotic yellow vein virus | 26.666666666666668 | 33.333333333333336 | 33.333333333333336 |
| Beet virus Q | 16.666666666666668 | 16.666666666666668 | 23.333333333333336 |
| Beta vulgaris mitovirus 1 | 96.66666666666667 | 100.0 | 100.0 |
| Melon chlorotic spot virus | 3.3333333333333335 | 0.0 | 3.3333333333333335 |
